# The effects of Saline Infusion Sonography on the histological quality of endometrial sampling in women with postmenopausal bleeding

**DOI:** 10.1186/s12905-023-02178-6

**Published:** 2023-02-04

**Authors:** Albertine J. Vroom, Simone Bongarts, Marlies Y. Bongers, Loes F. S. Kooreman, Steven L. Bosch, Peggy M. A. J. Geomini, Nehalennia van Hanegem

**Affiliations:** 1 Department of Gynecology and Obstetrics, Máxima Medisch Centrum, Veldhoven, The Netherlands; 2 Department of Obstetrics and Gynecology, GROW- School for Oncology and Reproduction, GMaastricht UMC+, P. Debyelaan 25, HX 6229 Maastricht, The Netherlands; 3 Department of Pathology, Maastricht UMC+, Maastricht, The Netherlands; 4 Laboratory for Pathology and Medical Microbiology (Stichting PAMM), Eindhoven, The Netherlands; 5 Department of Reproductive Medicine and Gynecology, University Medical Center, Utrecht, The Netherlands

**Keywords:** Postmenopausal bleeding, Endometrial biopsy, Endometrial pathology, Saline contrast sonohysterography

## Abstract

**Background:**

The aim of this study is to analyze the histopathological features of endometrial samples obtained by aspiration when performed before or after the saline contrast sonohysterography in women with postmenopausal bleeding and a thickened endometrium. Hypothetically, the saline infusion could disrupt the tissue and therefore affect the quality of the sample. Furthermore, we want to determine which histological features have impact on the quality of the endometrial sample.

**Methods:**

We performed a randomized controlled trial (ESPRESSO trial) in which we analyzed the aspiration samples in two groups. Women were allocated either to saline contrast sonohysterography and subsequent endometrial sampling (SCSH-Sampling group) or to the opposite order (Sampling-SCSH group). Dedicated gyneco-pathologists retrospectively assessed the specimens and recorded the type (blood, mucus, epithelium, intact glands, stroma and tissue context) and quantity (on a scale of 0–3) of material that was found in the specimens.

**Results:**

This analysis consisted of 197 samples, with 101 women in the SCSH-Sampling group and 96 women in the Sampling-SCSH group. No significant differences were found in the histological features between the two groups. All significant histological features differed significantly in the sufficient samples compared to the insufficient samples: higher amounts of blood, more endometrial epithelium, presence of intact endometrial glands, better stroma and tissue context. Oppositely, a significantly higher amount of mucus was found in the insufficient samples.

**Conclusion:**

This study shows that the histological features of the endometrial sample were not affected by the saline contrast sonohysterography, when performed prior to the tissue sampling.

*Trial registration* ESPRESSO TRIAL, NTR5690, registered 16 February 2016, https://trialsearch.who.int/Trial2.aspx?TrialID=NTR5690.

## Key massages

1. This secondary analysis of the randomized controlled ESPRESSO trial, to investigate the histological features of the endometrial sample obtained before or after the saline contrast sonohysterography in women with postmenopausal bleeding, showed that the histological features of the endometrial sample were not affected by the saline contrast sonohysterography.
2. This study also showed that a higher amount of blood, endometrial epithelium, intact endometrial glands, stroma and tissue context were found in sufficient endometrial samples compared to insufficient endometrial samples. Oppositely, a significantly higher amount of mucus was found in the insufficient samples.

## Background

Postmenopausal bleeding (PMB) can be the first clinical sign of endometrial pathology. Women with PMB and an endometrial thickness of more than four mm on ultrasound carry a potential high risk of an endometrial malignancy. The risk for an endometrial malignancy is about 10% in women with PMB and a thickened endometrium. The presence of endometrial polyps is reported in 40% of women with PMB and a thickened endometrium [1, 2]. These polyps carry a 4–6% risk for a focal (pre)malignancy [3, 4]. Because of this increased risk of malignancy, evaluation is advocated in all women with PMB [5].

The endometrial aspiration sample is an accurate test to detect endometrial malignancies, however focal lesions, such as polyps, can be missed [3]. To perform complete diagnostic work-up, national Dutch guidelines recommend endometrial sampling and a saline contrast sonohysterography (SCSH) if a previous transvaginal ultrasound shows an endometrial thickness of > 4 mm. [6]. Although current guidelines advise gynecologists to perform both procedures in their diagnostic work up, there is no consensus in which order the procedures should be performed. Hypothetically the quality of the sample can be affected by the saline used in the SCSH, when sampling is performed after the SCSH, because specific histological structures could be disrupted.

We performed a randomized controlled trial (ESPRESSO trial) in which we analyzed the quality of the aspiration samples in two groups: One group first received the SCSH and subsequently the endometrial aspiration sample, the second group first received the endometrial aspiration sample and subsequently the SCSH. The primary analysis of the ESPRESSO trial showed that the quality of the endometrial sample, classified as sufficient or insufficient following a standard protocol by an in-house pathologist, was not affected by whether SCSH was performed before or after endometrial sampling [7].

However, the quality of the endometrial sample is based on specific histological features. These features were not specifically analyzed in the primary results. For the current study, we assessed these specific histological features of the endometrial samples. We hypothesized that specific histological features of the endometrial samples would be affected by the SCSH. An essential histological feature to evaluate the endometrial aspiration sample based on expert opinions is the tissue context. This context could be disrupted by the saline infusion. We also hypothesized that the endometrial glands would be damaged due to the SCSH. Both factors would potentially affect the quality of the endometrial sample. Furthermore, we wanted to determine which histological features are critical for the quality of the endometrial sample by comparing the histological features of the sufficient and insufficient samples.

## Methods

Inclusion for the ESPRESSO trial (NTR5690) was offered to women presenting with PMB and a thickened endometrium of more than four mm at the department of Obstetrics and Gynecology in Máxima Medical Centre, Veldhoven, and Maastricht University Medical Centre, Maastricht, between April 2016 and February 2018. All details on the randomized controlled trial are described in the manuscript and study protocol of the ESPRESSO trial [7, 8]. Women receiving hormone therapy and women with cervical cancer were excluded. Randomization was performed with sealed envelopes, using block randomization with alternating blocks and 1:1 allocation. Women were allocated either to SCSH and subsequent endometrial sampling (SCSH-Sampling group) or to the opposite order (Sampling-SCSH group). Endometrial sampling was performed by inserting a Pipelle® device (Pipelle de Cornier, Paris, France) and an SCSH was performed using separate devices; a SCSH-catheter (Echosampler by Gynetics Medical devices, Lommel, Belgium). 232 women were randomized in the ESPRESSO trial, endometrial samples were obtained in 197 women. The samples were reported as sufficient or insufficient for diagnosis by an in-house pathologist. Sufficient means that the tissue was easily assessable and a histological diagnosis could be made; insufficient means that the tissue was not assessable and a histological diagnosis could not be made. For the current study, independent dedicated gyneco-pathologists, who were blinded for the order of intervention and primary diagnosis, retrospectively looked into the specimens of the endometrial samples. We choose to analyze all the 197 endometrial samples which were available for the primary analysis of the ESPRESSO trial. Three dedicated pathologists, from both participating medical centers, evaluated the samples and recorded the type and quantity (on a scale of 0–3) of material that was found in the specimens by using a specimen form designed for this study to score presence and amount of blood, mucus, epithelium, intact glands, stroma and tissue context. Additionally, the pathologist determined whether hyperplasia, atypia or malignancy were present. The results of this second evaluation had no consequences for the patient’s further (possible) treatment or investigations.

### Statistical analysis

Baseline characteristics are presented as median with inter quartile range (IQR) and number of patients (n, %). Histological features of the endometrial samples of the SCSH-Sampling and the Sampling-SCSH group were compared using a Mann-Whitney U test. Furthermore, histological features of the sufficient and insufficient samples were compared to determine which features are critical for the quality (whether the sample was sufficient or insufficient) of the samples.

For statistical analysis, the Statistical Package for the Social Sciences (IBM Corp, Armonk, NY, USA) version 24.0 was used. Statistical significance was set at p < 0.05.

## Results

This analysis consisted of 197 samples, with 101 women in the SCSH-Sampling group and 96 women in the Sampling-SCSH group. Patient characteristics are shown in Table 1.

**Table 1:** Baseline characteristics

	SCSH-sampling n = 101	Sampling-SCSH n = 96
Age – Median (IQR)	58 (54–67)	59 (55–65)
BMI (kg/m2) - Median (IQR)	28.4 (24.2–33.8)	29.5 (26.4–34.0)
Parity - Median (IQR) Vaginal birth	2 (1–3)	2 (2–3)
	2 (2–3)	2 (2–3)
Months PMP - Median (IQR)	47 (18–184)	73 (20–140)
No. of samples with hyperplasia without atypia – n (%)	5 (5)	5 (5)
No. of samples with atypia – n (%)	6 (6)	8 (8)
No. of samples with malignancy n (%)	8 (8)	10 (10)

BMI = body mass index; IQR = interquartile range; PMP = postmenopausal SCSH = saline contrast sonohysterography

Comparison of histologic findings between the SCSH-Sampling and Sampling-SCSH group is shown in Table 2. No significant differences were found in the histological features between the two groups. The extend of the presence of the specific histological features, e.g. the amount of intact endometrial glands, was comparable between the two groups.

**Table 2:** Comparison of histologic findings between SCSH-Samplingand Sampling-SCSH group. Values are presented as number (%)

	SCSH-sampling (n = 101)	Sampling-SCSH (n = 96)	*P*
*Amount of blood*			0.08
Not present Little Moderate A lot	34 (34%) 27 (27%) 16 (16%) 24 (24%)	42 (44%) 26 (27%) 13 (14%) 15 (16%)	
*Amount of mucus*			0.88
Not present Little Moderate A lot	35 (35%) 32 (32%) 16 (16%) 18 (18%)	33 (34%) 32 (33%) 16 (17%) 15 (16%)	
*Amount of endometrial epithelium*			0.06
Not present Little Moderate A lot	7 (7%) 34 (34%) 26 (26%) 34 (34%)	5 (5%) 21 (22%) 28 (29%) 42 (44%)	
*Amount of intact endometrial glands*			0.20
Not present Little Moderate A lot	32 (32%) 21 (21%) 24 (24%) 24 (24%)	24 (25%) 21 (22%) 18 (18%) 32 (33%)	
*Amount of stroma*			0.13
Not present Little Moderate A lot	22 (22%) 27 (27%) 24 (24%) 28 (28%)	13 (14%) 25 (26%) 25 (26%) 33 (34%)	
*Amount of tissue context*			0.14
Not present Little Moderate A lot	33 (33%) 20 (20%) 24 (24%) 24 (24%)	27 (28%) 14 (15%) 22 (23%) 33 (34%)	

Table 3 shows the results of the comparison of the sufficient versus the insufficient samples.

**Table 3:** Comparison of histologic findings between the sufficient versus the insufficient samples. Values are presented as number (%)

	Insufficient (n = 62)	Sufficient (n = 135)	*P*
*Amount of blood*			0.00
Not present Little Moderate A lot	36 (58%) 12 (19%) 7 (11%) 7 (11%)	40 (30%) 41 (30%) 22 (16%) 32 (24%)	
*Amount of mucus*			0.00
Not present Little Moderate A lot	12 (19%) 23 (37%) 7 (11%) 20 (32%)	56 (41%) 41 (30%) 25 (19%) 13 (10%)	
*Amount of endometrial epithelium*			0.00
Not present Little Moderate A lot	12 (19%) 33 (53%) 11 (18%) 6 (10%)	0 (0%) 22 (16%) 43 (32%) 70 (52%)	
*Amount of intact endometrial glands*			0.00
Not present Little Moderate A lot	41 (66%) 16 (26%) 3 (5%) 2 (3%)	15 (11%) 26 (19%) 39 (29%) 54 (40%)	
*Amount of stroma*			0.00
Not present Little Moderate A lot	30 (48%) 21 (34%) 8 (13%) 3 (5%)	5 (4%) 31 (23%) 41 (30%) 58 (43%)	
*Amount of tissue context*			0.00
Not present Little Moderate A lot	46 (74%) 9 (15%) 6 (10%) 1 (2%)	14 (10%) 25 (19%) 40 (30%) 56 (41%)	

A significant difference was found for all the histological features with higher amounts of blood, endometrial epithelium, intact endometrial glands, stroma and tissue context in the sufficient samples. Oppositely, a significantly higher amount of mucus was found in the insufficient samples.

Table 4 shows the results of the comparison of the 135 sufficient samples between the SCSH-Sampling and Sampling-SCSH group. No significant differences were found in almost all the histological features between the two groups. Only a significantly higher amount of blood was found in the SCSH-Sampling group.

**Table 4:** Comparison of histologic findings between SCSH-Samplingand Sampling-SCSH group for the sufficient samples. Values are presented as number (%)

	SCSH-sampling (n = 65)	Sampling-SCSH (n = 70)	*P*
*Amount of blood*			**0.03**
Not present Little Moderate A lot	15 (23%) 19 (29%) 10 (15%) 21 (32%)	25 (36%) 22 (31%) 12 (17%) 11 (16%)	
*Amount of mucus*			0.88
Not present Little Moderate A lot	26 (40%) 21 (12%) 12 (19%) 6 (9%)	30 (43%) 20 (29%) 13 (19%) 7 (10%)	
*Amount of endometrial epithelium*			0.15
Not present Little Moderate A lot	0 (0%) 15 (23%) 19 (29%) 31 (48%)	0 (0%) 7 (10%) 24 (34%) 39 (56%)	
*Amount of intact endometrial glands*			0.30
Not present Little Moderate A lot	9 (14%) 12 (19%) 21 (32%) 23 (35%)	6 (9%) 14 (20%) 18 (26%) 31 (44%)	
*Amount of stroma*			0.59
Not present Little Moderate A lot	3 (5%) 16 (25%) 19 (29%) 27 (42%)	2 (3%) 15 (21%) 22 (31%) 31 (44%)	
*Amount of tissue context*			0.18
Not present Little Moderate A lot	8 (12%) 13 (20%) 21 (32%) 23 (35%)	6 (9%) 12 (17%) 19 (27%) 33 (47%)	

Bold indicates that the statistical significance at *p* < 0.05

Table 5 shows the results of the comparison of the 62 insufficient samples between the SCSH-Sampling and Sampling-SCSH group. In this group, the extend of the presence of the specific histological features, e.g. the amount of intact endometrial glands, was comparable between the two groups as well.

**Table 5:** Comparison of histologic findings between SCSH-Samplingand Sampling-SCSH group for the insufficient samples. Values are presented as number (%)

	SCSH-sampling (n = 36)	Sampling-SCSH (n = 26)	*P*
*Amount of blood*			0.46
Not present Little Moderate A lot	9 (25%) 11 (31%) 4 (11%) 12 (33%)	3 (12%) 12 (46%) 3 (12%) 8 (31%)	
*Amount of mucus*			0.70
Not present Little Moderate A lot	9(25%) 11(31%) 4(11%) 12(33%)	3 (12%) 12 (46%) 3 (12%) 8 (31%)	
*Amount of endometrial epithelium*			0.98
Not present Little Moderate A lot	7 (19%) 19 (53%) 7 (19%) 3 (8%)	5 (19%) 14 (54%) 4 (15%) 3 (12%)	
*Amount of Intact endometrial glands*			0.57
Not present Little Moderate A lot	23 (64%) 9 (25%) 3 (8%) 1 (3%)	18 (69%) 7 (27%) 0 (0%) 1 (4%)	
*Amount of stroma*			0.44
Not present Little Moderate A lot	19 (53%) 11 (31%) 5 (14%) 1 (3%)	11 (42%) 10 (38%) 3 (12%) 2 (8%)	
*Amount of tissue context*			0.37
Not present Little Moderate A lot	25 (69%) 7 (19%) 3 (8%) 1 (3%)	21 (81%) 2 (8%) 3 (12%) 0 (0%)	

No new cases of atypia or malignancy were detected at this pathology review by the dedicated gyneo-pathologists.

## Discussion

No significant differences were found in the histological features of the samples when the endometrial sample was obtained before or after the SCSH. In the sub analysis of only sufficient samples, also no significant differences were found in the histological features apart form a higher amount of blood which was found in the SCSH-Sampling group.

Therefore, we can conclude the SCSH does not affect the histological features, and thus the quality, of the endometrial samples.

This study also shows that a higher amount of blood, endometrial epithelium, intact endometrial glands, stroma and tissue context were found in the sufficient samples compared to the insufficient samples. Oppositely, a significantly higher amount of mucus was found in the insufficient samples.

These results are in line with the results of the primary analysis of the ESPRESSO RCT, which concluded that the quality of the endometrial sample was not affected by whether SCSH was performed before or after endometrial sampling. However, this primary analysis only focused on general adequacy of the sampling and did not assess specific histological features of the endometrial sample such as amount of epithelium, intact glands and stroma. As we were still interested in our predefined hypothesis, we aimed to analyze whether the effect of the SCSH would be seen in the specific histological features of the endometrial sample [7].

In addition, we wanted to determine which histological features have impact on the quality of the endometrial sample. This is of clinical interest, as insufficient endometrial samples have been a common problem in the diagnostic pathway in women with PMB with a reported rate of 7–78% of insufficient samples [9, 10]. Previous research to decrease the amount of samples rated as insufficient by developing a structured assessment did not seem feasible [9]. However, in this previous assessment, the samples were only scored on estimated amount of material and the amount of histopathological endometrial tissue.

Our study suggests that multiple factors determine the quality of the endometrial samples as higher amounts of blood, endometrial epithelium, intact endometrial glands, stroma and tissue context were found in the sufficient samples compared to the insufficient samples. Based on these results, further research to develop a more extensive structured assessment of the endometrial samples in women with PMB, is interesting.

Also, the amount of tissue is an important factor and very little material in the sample could lead to diagnostic errors. For this reason, it would be advisable for a gynecologist to macroscopically check the amount of tissue in the container after sampling and consider to reinsert the device to take another sample if only very little tissue, or only mucus, has been sampled.

Furthermore, It also has to be noted that the clinical patient characteristics, such as hormonal replacement therapy and the thickness of the endometrium on ultrasound, are valuable details for the pathologist in the assessment of the aspiration sample [11]. These characteristics should be taken in consideration in the development of an extensive structured assessment of the endometrial sample and can be a subject for future research.

It is remarkable that the amount of blood was significantly higher in the group of sufficient samples. Blood and mucus are seen as staining of the endometrial sample by the pathologist, and some would define an inadequate sample if consisting only of blood or cervical mucus with fragments of benign endocervix, or a large amount of blood with only small fragments of endometrial glands and stroma [12]. The results of this study show that a high amount of blood does not affect the quality of the endometrial sample and therefore a sample should not be classified by the amount of blood.

Bij de Vaate performed a randomized controlled trial to compare the quality and histological features of an endometrial sample obtained before or after the SCSH in predominantly pre-menopausal women [13]. Our results are not in concordance with this previous study as this study showed a significant difference in the amount of endometrial epithelium when the sample was performed after the SCSH. The histological composition of the endometrium in premenopausal women is different compared to the endometrium in postmenopausal women and this difference could explain the fact that our results are not in concordance.

This is a secondary analysis of the randomized controlled trial to investigate the histological features of the endometrial sample obtained before or after the SCSH in women with PMB. The samples were analyzed thoroughly and structurally by dedicated gyneco-pathologists. A limitation of the current study was that there was no sample size calculation performed, as this was a secondary analysis.

## Conclusion

This study shows that the histological features of the endometrial sample were not affected by the saline contrast sonohysterography.

## Data Availability

The datasets generated and analyzed during the current study are available from the corresponding author on reasonable request.

## Abbreviations

PMB: Postmenopausal bleeding
SCSH: Saline contrast sonohysterography
IQR: Inter quartile range
